# Antibodies to mutated citrullinated vimentin for diagnosing rheumatoid arthritis in anti-CCP-negative patients and for monitoring infliximab therapy

**DOI:** 10.1186/ar2570

**Published:** 2008-12-10

**Authors:** Pascale Nicaise Roland, Sabine Grootenboer Mignot, Alessandra Bruns, Margarita Hurtado, Elisabeth Palazzo, Gilles Hayem, Philippe Dieudé, Olivier Meyer, Sylvie Chollet Martin

**Affiliations:** 1 Immunology and Haematology Department, Bichat-Claude Bernard Teaching Hospital, AP-HP, 46, rue H Huchard 75877 Paris Cedex 18, France; 2 Rheumatology Department, Bichat-Claude Bernard Teaching Hospital, AP-HP, 46 rue H Huchard 75877 Paris Cedex 18, France; 3 Inserm IFR 141, UMR756, Paris-South 11 University, 5 rue JB Clement 92296 Chatenay-Malabry Cedex, France

## Abstract

**Introduction:**

Antibodies against cyclic citrullinated peptides (CCPs) are useful for diagnosing rheumatoid arthritis (RA). Antibodies to mutated citrullinated vimentin (MCV) were described recently in RA. The aims of this study were to evaluate the usefulness of anti-MCV for diagnosing RA in anti-CCP-negative patients and to monitor anti-MCV titres during infliximab therapy for RA.

**Methods:**

We studied two groups of RA patients, one with (n = 80) and one without (n = 76) anti-CCP antibodies. The specificity of anti-MCV was evaluated by investigating 50 healthy controls and 158 patients with other rheumatic diseases (51 psoriatic rheumatism, 58 primary Sjögren syndrome, and 49 ankylosis spondylitis). Serum anti-MCV and anti-CCP titres were measured in 23 patients after 6, 12, 18, and 24 months of infliximab treatment. Anti-CCP2 and anti-MCV levels were assayed using a commercial enzyme-linked immunosorbent assay. IgM rheumatoid factor was determined by nephelometry.

**Results:**

In accordance with the cutoff values recommended by the manufacturer, the specificity of anti-MCV antibodies was 90.9%. We adjusted the cutoff values to obtain the same specificity as that of anti-CCP antibodies (94.2%). With this optimal cutoff, anti-MCV antibodies were found in 11.8% (9/76) of RA patients without anti-CCP, and similarly, anti-CCP antibodies were found in 11.2% (9/80) of RA patients without anti-MCV. Anti-MCV antibodies were positive in 6 patients who tested negative for both anti-CCP and rheumatoid factor. Anti-MCV titres were significantly decreased after 18 and 24 months of infliximab therapy compared with baseline (*P *< 0.01) as a significant decrease of anti-CCP levels occurred only at 24 months (*P *< 0.04). Moreover, an anti-MCV decrease was significantly associated with DAS28 (disease activity score using 28 joint counts) improvements 12 months into therapy.

**Conclusions:**

Our results suggest that anti-MCV antibodies may be valuable for diagnosing RA in anti-CCP-negative patients without replacing them as an equivalent number of anti-CCP-positive RA patients test negative for anti-MCV. Moreover, anti-MCV antibodies could be useful for monitoring the effects of infliximab therapy.

## Introduction

Rheumatoid arthritis (RA) is the most common chronic inflammatory joint disease, with a worldwide prevalence of 0.5% to 1%. RA is characterized by synovial joint inflammation, which often leads to progressive joint destruction and disability [1]. Early treatment improves the outcome and therefore early diagnosis is crucial. Rheumatoid factors (RFs) were the first biological markers discovered for RA and remain the only laboratory criterion included in the American College of Rheumatology criteria for RA classification [2]. Two major disadvantages of RF are low specificity and possible absence in the first year of the disease [3]. Several other auto-antibodies specific to RA have been found. Among them, anti-filaggrin antibodies, anti-keratin antibodies (AKAs), and anti-perinuclear factor (APF) exhibit a high specificity and sometimes present early in the disease. However, AKA is not sufficiently sensitive to be used for diagnosing RA. APF detection is available only at specialized laboratories, as obtaining and standardizing the substrate is technically challenging, and interpreting the immunofluorescence results is largely subjective [4].

Anti-filaggrin antibodies recognize citrulline residues formed by post-transcriptional modification of arginine by peptidylarginine deiminase [5]. Enzyme immunoassays (EIAs) based on synthetic cyclic citrullinated peptides (CCP2) are available for detecting anti-CCP. We and others previously showed that the sensitivity of these antibodies was about 80% in established RA [6,7] compared with 55% in early RA [6,8] and 40% in very early RA [9,10]. The usefulness of anti-CCP for monitoring RA patients, particularly during treatment, is controversial. A significant decrease was found after 6 months of tumor necrosis factor (TNF) antagonist therapy in one study [11], whereas decreases were slow and inconsistent during infliximab therapy in two other studies [12,13].

Antibodies to other citrullinated peptides or proteins have been suggested as good candidates for diagnosing RA. Vimentin is an intermediate filament that is widely expressed by mesenchymal cells and macrophages and easy to detect in the synovium. Modification of the protein occurs in macrophages undergoing apoptosis, and antibodies to citrullinated vimentin may emerge if the apoptotic material is inadequately cleared [14]. The first antibodies to citrullinated vimentin described in the literature were anti-Sa antibodies detected by Western blot, which were as specific as anti-CCP but not sufficiently sensitive (20% to 45%) to serve as diagnostic tools [15,16]. Recombinant mutated citrullinated vimentin (MCV) was recently produced, and an enzyme-linked immunosorbent assay (ELISA) was developed for detecting anti-MCV. Few data are available on the performance of anti-MCV for diagnosing RA. In most studies, anti-MCV and anti-CCP testing produced similar results [17-20]. Two studies, however, suggested that anti-MCV might be more sensitive than anti-CCP [21,22].

The aims of this study were to evaluate the usefulness of anti-MCV for diagnosing RA in anti-CCP-negative patients and to monitor anti-MCV titres during infliximab therapy for RA. First, we compared the results of anti-MCV tests in RA patients with and without positive tests for anti-CCP. Then, we obtained serial anti-MCV assays in RA patients receiving infliximab therapy.

## Materials and methods

### Patients

We studied 156 patients seen at the Rheumatology Department of the Bichat-Claude Bernard Teaching Hospital (Paris, France) for RA meeting American College of Rheumatology (ACR) criteria. Among them, 20 had early RA (<12 months). The control group was composed of 50 healthy controls and 158 patients seen at the same department for other diseases, including psoriatic arthritis (n = 51), primary Sjögren syndrome (n = 58), and ankylosing spondylitis (n = 49).

Among the RA patients, 23 were evaluated after 6, 12, 18, and 24 months of infliximab therapy. All 23 patients had a history of inadequate response or tolerance to at least one conventional disease-modifying antirheumatic drug (DMARD). Previous treatment with DMARDs (methotrexate, hydroxychloroquine, azathioprine, or sulfasalazine), steroids, and nonsteroidal anti-inflammatory drugs could be continued provided that the dosage was kept stable for at least 4 weeks before infliximab initiation and throughout infliximab therapy. Infliximab was administered (3 mg/kg) at baseline, 2 and 6 weeks later, and every 8 weeks thereafter in combination with a DMARD. None of the patients had active or latent tuberculosis, other infections, or severe comorbidities.

The following variables were collected at baseline and then after 3, 6, 12, 18, and 24 months: tender and swollen joint counts of a total of 28 joints, score on a visual analog scale for global health completed by the patient, erythrocyte sedimentation rate, and C-reactive protein level. The disease activity score using 28 joint counts (DAS28) was computed at 0, 6, and 12, months; the amount of missing data was too large for reliable DAS28 determination at the 3-month time point. Responders were defined at different time points as having a DAS28 decrease of greater than 1.2 with a DAS28 value of less than 3.2, and nonresponders were defined as having a DAS28 decrease of less than 0.6 or a decrease in the 0.6 to 1.2 range with a score value of greater than 5.1 [23]. Data for responders and nonresponders were compared only at 6 and 12 months as the number of nonresponder patients available at 18 and 24 months was too low for a statistical analysis.

The patients were informed of the purpose of the study and gave their informed consent. The trial was approved by the ethics committee of our hospital. All procedures were conducted in accordance with the hospital's ethics rules. All the sera were stored at -20°C until the assays.

### Methods

IgM-RF was determined by nephelometry using a BNII analyser (N RF Latex; Dade Behring, Paris La Défense, France). Levels greater than 20 IU/mL were considered positive. Anti-CCP2 was assayed using an EIA (Immunoscan RA; Euro-Diagnostica, Arnhem, The Netherlands) in accordance with the instructions of the manufacturer. Titres lower than 25 units were considered negative. Anti-MCV levels were determined using a commercial ELISA (Orgentec Diagnostika, Mainz, Germany) with MCV as the antigen. Briefly, sera diluted 1:100 were incubated for 30 minutes on the coated plate, which was then washed before the addition of horseradish peroxidase-labelled goat anti-human IgG for 15 minutes. The reaction was revealed by the addition of TMB (3,3', 5,5'-tetramethylbenzidine) substrate, and color intensity was measured at 450/620 nm. Values greater than 20 U/mL were considered positive, which was in accordance with the instructions of the manufacturer.

### Statistical analysis

The chi-square test was used to compare the specificities of anti-MCV, anti-CCP, and IgM-RF. The nonparametric Wilcoxon signed rank test was used for paired comparisons of changes in anti-CCP and anti-MCV titres during infliximab treatment for all of the RA patients as well as after a separation between responders and nonresponders. The Mann-Whitney test was used to compare anti-CCP, anti-MCV, and IgM-RF titres at baseline in responders and nonresponders. The Spearman rank correlation test was used to study the correlation between the DAS28 and anti-CCP or anti-MCV titres at baseline. A *P *value of 0.05 or less was considered statistically significant.

## Results

### Specificity of anti-mutated citrullinated vimentin for rheumatoid arthritis

The prevalence of anti-MCV, anti-CCP, and IgM-RF in the 208 controls is reported in Table 1. Thus, the specificity of anti-MCV was 90.9%, in accordance with the cutoff values recommended by the manufacturer, in comparison with 94.7% for anti-CCP antibodies. We adjusted the cutoff value of anti-MCV (28 U/mL) to obtain the same specificity as that of anti-CCP antibodies. Anti-CCP and anti-MCV antibodies were significantly more specific than IgM-RF (89.9%, 85.8% to 94%; *P *< 0.05). Of the 12 controls who had positive anti-MCV titres, 5 had psoriatic arthritis, 5 primary Sjögren syndrome, and 2 ankylosing spondylitis. Among the 5 patients who had psoriatic arthritis and positive anti-MCV titres, only 1 had negative results for the other two markers and 3 had positive results for both anti-CCP and RF. Among the 5 patients with primary Sjögren syndrome and positive anti-MCV titres, only 1 had negative results for the other two markers and 4 had positive results for both of these markers. Of the 2 patients with ankylosing spondylitis and positive anti-MCV titres, 1 had positive anti-CCP titres.

**Table 1:** Anti-MCV, anti-CCP, and IgM-RF antibodies in the control patients according to the diagnosis

Number (percentage) of patients with positive antibodies				
	Anti-MCV		Anti-CCP	IgM-RF
			
	Cutoff 20 U/mL	Cutoff 28 U/mL		
Psoriatic arthritis (n = 51)	6 (11.8)	5 (9.8)	5 (9.8)	5 (9.8)
Primary Sjögren syndrome (n = 58)	6 (10.3)	6 (10.3)	6 (10.3)	15 (25.8)
Ankylosing spondylitis (n = 49)	3 (6.1)	2 (4.1)	1 (2)	1 (2)
Healthy controls (n = 50)	5 (10)	0 (0)	0 (0)	0 (0)
Total (n = 208)	20 (9.6)	13 (6.2)	12 (5.8)	21 (10.1)

Anti-CCP, anti-cyclic citrullinated peptide antibody; anti-MCV, anti-mutated vimentin citrullinated antibody; RF, rheumatoid factor.

### Comparison of rheumatoid arthritis patients with and without anti-cyclic citrullinated peptide

Of the 156 patients with RA, 80 had positive anti-CCP titres and 76 had negative anti-CCP titres. Table 2 reports the results of the anti-MCV and IgM-RF assays in these two groups at the different cutoffs. Of the 80 RA patients with anti-CCP, 71 (88.7%) were anti-MCV-positive, including 10 (12.5%) who were IgM-RF-negative. Of the 9 (11.2%) anti-CCP-positive patients without anti-MCV, 4 also tested negative for IgM-RF. Of the 76 RA patients without anti-CCP, 9 (11.8%) were positive for anti-MCV, including 6 who tested negative for IgM-RF. Thus, in these 6 patients, anti-MCV was the only positive marker, indicating that anti-MCV assays may help to diagnose RA in patients with negative tests for anti-CCP and IgM-RF. The presence of anti-MCV was confirmed in another blood sample 1 year later. Five of the 9 RA patients positive for anti-MCV and negative for anti-CCP had low levels of anti-MCV (between 30 and 35 U/mL). We could not identify a characteristic clinical profile of those patients as 2 had a deforming RA, 1 an erosive RA, and 3 a more benign form of the disease. Of the 80 RA patients who tested positive for anti-CCP, 7 (8.7%) had early disease and 6 tested positive for anti-MCV with the optimal cutoff. Of the 76 RA patients who tested negative for anti-CCP, 13 (17.1%) had early RA, including 3 who tested positive for anti-MCV. The number of patients with early RA in our study was too small for a statistical evaluation of these data.

**Table 2:** Distribution of the rheumatoid arthritis patients according to the presence of anti-MCV, anti-CCP, and IgM-RF

	Anti-CCP-positive (n = 80), number (percentage)		Anti-CCP-negative (n = 76), number (percentage)	
		
	Cutoff 20 U/mL	Cutoff 28 U/mL	Cutoff 20 U/mL	Cutoff 28 U/mL
Anti-MCV				
Positive	75 (93.75)	71 (88.75)	14 (18.4)	9 (11.8)
Negative	5 (6.25)	9 (11.25)	62 (81.6)	67 (88.2)

RF^+^				
Anti-MCV^+^	65 (81.25)	61 (76.25)	5 (6.6)	3 (3.9)
Anti-MCV^-^	1 (3.75)	5 (6.25)	8 (10.5)	10 (13.2)

RF^-^				
Anti-MCV^+^	10 (12.5)	10 (12.5)	9 (11.8)	6 (7.9)
Anti-MCV^-^	4 (5)	4 (5)	54 (71.1)	57 (75)

Anti-CCP, anti-cyclic citrullinated peptide antibody; anti-MCV, anti-mutated vimentin citrullinated antibody; RF, rheumatoid factor.

### Changes in anti-mutated citrullinated vimentin titres during infliximab therapy

**Figure 1:**
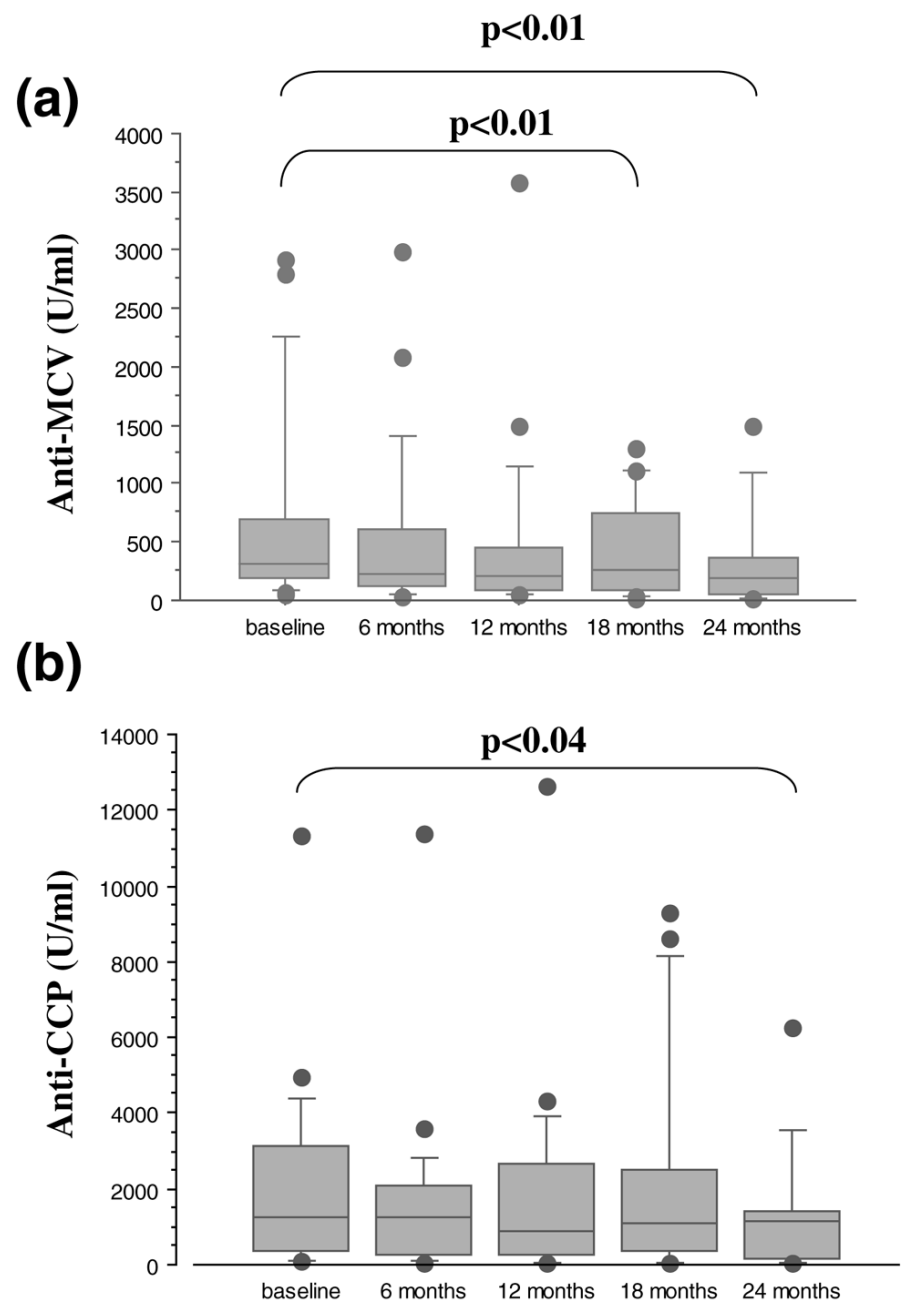
Changes in anti-MCV **(a) **and anti-CCP **(b) **titres during infliximab treatment. Values are presented as box-and-whisker plots, in which each box represents the interquartile range, the line within the box represents the median, the whiskers represent 10th to 90th percentiles of the titres, and the circles represent the range from the smallest to the largest value. The nonparametric Wilcoxon signed rank test was used for paired comparisons of changes in anti-CCP and anti-MCV titres. CCP, cyclic citrullinated peptide; MCV, mutated citrullinated vimentin.

Of the 80 patients with anti-CCP antibodies, 23 started infliximab therapy and were subsequently followed for at least 24 months. At baseline, all patients had active disease as assessed by the DAS28 (mean of 5.3 ± 0.8). Anti-MCV levels did not correlate to the DAS28 at baseline (*r *= 0.191, Spearman rank correlation test), whereas the correlation coefficient was better than between anti-CCP and DAS28 (*r *= -0.01). Figure 1 reports changes in anti-CCP and anti-MCV titres during infliximab therapy, and the nonparametric Wilcoxon signed rank test was used for paired comparisons of changes in anti-CCP and anti-MCV titres. Significant decreases occurred in anti-MCV titres at 18 and 24 months (*P *< 0.01) and in anti-CCP titres at 24 months (*P *< 0.04). Their faster decrease may make anti-MCV more useful than anti-CCP for monitoring infliximab therapy. Therefore, we looked for an association between antibody decreases and the DAS28 response. The numbers of DAS28 responders were 11 after 6 months and 15 after 12 months. Anti-MCV, anti-CCP, and IgM-RF titres at baseline were not significantly different between DAS28 responders and nonresponders (Mann-Whitney test). After 12 months, in contrast, the anti-MCV titre decrease was significant in the group of responders but not in the group of nonresponders compared by the Wilcoxon signed rank test (Table 3). The same was true of anti-CCP titres, although the difference was less marked. The median titres of anti-CCP in nonresponders were apparently higher at 12 months compared with baseline, but the individual paired analysis suggested a 4% decrease for each individual patient.

**Table 3:** Anti-MCV, anti-CCP, and IgM-RF at baseline and after 6 and 12 months of infliximab therapy

	Response 6 months	Baseline	6 months	Response 12 months	Baseline	12 months
Anti-MCV, U/mL	Responders (n = 11)	654 (89–2,918)	535 (56–3,000)	Responders (n = 15)	293 (61–2,918)	117^a ^(48–3,582)
	Nonresponders (n = 12)	307 (60–1,145)	227 (40–1,234)	Nonresponders (n = 8)	363 (60–1,145)	227 (47–1,500)
Anti-CCP, U/mL	Responders (n = 11)	1,402 (83–11,360)	1,300 (54–11,370)	Responders (n = 15)	1,246 (83–11,360)	837^b ^(48–12,640)
	Nonresponders (n = 12)	962 (130–4,232)	1,134 (74–2,392)	Nonresponders (n = 8)	1,169 (275–4,232)	1,331 (126–4,313)
IgM-RF, IU/mL	Responders (n = 11)	91.5 (10–424)	58 (10–216)	Responders (n = 15)	74.5 (9–781)	49.5^c ^(10–214)
	Nonresponders (n = 12)	205 (9–949)	133^c ^(10–367)	Nonresponders (n = 8)	283 (14–949)	152^c ^(35–463)

Values are expressed as median (range), and the nonparametric Wilcoxon signed rank test was used for paired comparisons of changes in anti-CCP and anti-MCV titres between responders and nonresponders. ^a^*P *< 0.01 versus baseline; ^b^*P *< 0.05 versus baseline; ^c^*P *= 0.02 versus baseline. Anti-CCP, anti-cyclic citrullinated peptide antibody; anti-MCV, anti-mutated vimentin citrullinated antibody; RF, rheumatoid factor.

## Discussion

We evaluated the interest of antibodies to the citrullinated protein MCV in anti-CCP negative patients who met ACR criteria for RA. At comparable specificity, of the 63 patients with negative tests for both anti-CCP and RF, 6 (7.9%) were positive for anti-MCV. Moreover, anti-MCV titres decreased faster than did anti-CCP during infliximab therapy and showed a closer association with the DAS28 response.

Anti-CCP assays are effective and widely used for diagnosing RA. However, their sensitivity is limited to 40% in patients with early RA [9,10]. CCP is not expressed in the synovium, and citrullinated proteins expressed in the rheumatoid joint would probably be more relevant as targets of auto-antibodies used to diagnose RA. Citrullinated vimentin is present in synovial membranes and is released in increased amounts in response to growth factors and proinflammatory cytokines, suggesting involvement in the pathophysiology of RA [24]. The few studies of anti-MCV usually found performance characteristics similar to those of anti-CCP for diagnosing RA [17-20]. For Bang and colleagues [21], anti-MCV antibodies were even more sensitive than anti-CCP.

The most useful characteristic of anti-CCP in clinical practice is high specificity for RA. Therefore, we assessed the specificity of anti-MCV in controls and a large group of patients with established inflammatory rheumatic diseases. At the cutoff values recommended by the manufacturer, the specificity was lower than that of anti-CCP, as previously reported [20,25], and we adjusted the cutoff to obtain the same specificity as that of anti-CCP. Longer follow-up will determine whether positive anti-MCV assays, observed in several non-RA patients, predict subsequent RA as shown for anti-CCP [26,27].

An important finding from our study is that 11.8% of anti-CCP-negative RA patients were positive for anti-MCV. Furthermore, in 7.9% of our patients meeting ACR criteria for RA but having negative tests for anti-CCP, anti-MCV antibodies were positive although RF was negative. Similarly, 13.2% of anti-CCP-negative patients who also tested negative for anti-MCV had positive RF titres. In contrast, our results showed that an equivalent number of anti-CCP-positive RA patients (11.2%) tested negative for anti-MCV and among them 5% were also RF-negative. Therefore, the best diagnostic strategy may be to assay both anti-MCV and RF in anti-CCP-negative RA patients without replacing them. Of the 13 patients who had early RA and negative tests for anti-CCP, 3 had anti-MCV antibodies, which is in keeping with previous evidence that anti-MCV may help to improve the early diagnosis of RA [22].

We found that 9 anti-CCP-negative patients tested positive for anti-MCV (among them, 3 also tested positive for RF). Follow-up of this subgroup will determine whether anti-CCP antibodies emerge later on and whether the anti-CCP-negative anti-MCV-positive profile is of prognostic significance as suggested by Mathsson and colleagues [22]. Indeed, a recent report suggests that anti-MCV antibodies are better predictors of disease activity than anti-CCP [28]. Conceivably, the anti-CCP-negative anti-MCV-positive profile may be associated with specific gene polymorphisms. An association between the presence of RF and the mutation of the protein tyrosine phosphatase nonreceptor type 22 (*PTPN22*) gene has been evidenced [29]. Anti-CCP antibodies were associated with the *PTPN22 *1858 C/T polymorphism [30] and the HLADRB1 allele shared epitope [31].

Monitoring auto-antibody titres has been suggested as a means of assessing treatment efficacy. Several studies evaluated the effect of TNF antagonist treatment on anti-CCP titres in RA patients. A significant decrease was found after 6 months of TNF antagonist therapy in one study [11], whereas decreases were slow and inconsistent during infliximab therapy in two other studies [12,13]. Thus, anti-CCP seems to be of limited value for monitoring the effect of TNF antagonist therapy. Our study provides the first data on changes in anti-MCV titres during infliximab therapy for RA. We found a significant decrease in anti-MCV titres, which antedated the anti-CCP decrease; the former was first significant after 18 weeks and the latter after 24 weeks. Moreover, the anti-MCV decrease was related to the DAS28 response after 12 months, which is not the case for IgM-RF as their decrease is significant in responder as well as nonresponder RA patients. Previous reports showed a decrease of IgM-RF during anti-TNF treatment [11-13]. Association to clinical improvement was reported [12,32] at 6 months based essentially on the ACR response and not on the DAS28 response.

Most of the patients treated with infliximab in our study had long-standing RA. A disease duration of less than 1 year predicted a greater reduction in anti-CCP antibody titres with conventional treatment [33], suggesting that a study specifically designed to monitor anti-MCV titres in patients with early RA treated with TNF antagonists might be of interest. However, a recent report of Ursum and colleagues [34] found a very low correlation between the DAS28 and anti-MCV levels during a 2-year follow-up of early RA, suggesting that other parameters of the response are involved and should be studied.

## Conclusion

Our results suggest that anti-MCV and anti-CCP are complementary to enhance sensitivity of RA diagnosis as anti-MCV antibodies were found in 11.8% of RA patients without anti-CCP and that an equivalent number of anti-CCP-positive RA patients tested negative for anti-MCV. Moreover, during infliximab treatment, anti-MCV titres decreased more rapidly than anti-CCP titres and were associated with the DAS28 response, suggesting that they are helpful for monitoring infliximab therapy.

## Abbreviations

ACR: American College of Rheumatology; AKA: anti-keratin antibody; APF: anti-perinuclear factor; CCP: cyclic citrullinated peptide; DAS28: disease activity score using 28 joint counts; DMARD: disease-modifying antirheumatic drug; EIA: enzyme immunoassay; ELISA: enzyme-linked immunosorbent assay; MCV: mutated citrullinated vimentin; PTPN22: protein tyrosine phosphatase nonreceptor type 22; RA: rheumatoid arthritis; RF: rheumatoid factor; TNF: tumor necrosis factor.

## Competing interests

The authors declare that they have no competing interests.

## Authors' contributions

PNR contributed to the conception of the study and to the analysis and interpretation of data and was the main contributor to the preparation of the manuscript. SGM contributed to the acquisition and analysis of the data and to the preparation of the manuscript. AB contributed to the analysis of the data from infliximab-treated patients. MH contributed to the acquisition and analysis of the data. EP, GH, and PD were clinical investigators who contributed to the recruitment and treatment of the patients. OM was the main clinical investigator and contributed to the recruitment of the patients and to the critical review of the manuscript. SCM contributed to the interpretation of data and to the critical review of the manuscript. All authors read and approved the final manuscript.
